# Evaluation of Design Method for Highway Adjacent Tunnel and Exit Connection Section Length Based on Entropy Method

**DOI:** 10.3390/e24121794

**Published:** 2022-12-08

**Authors:** Yutong Liu, Binghong Pan, Zelong Zhang, Ranyang Zhang, Yang Shao

**Affiliations:** 1 Highway Academy, Chang’an University, Xi’an 710064, China; 2 School of Modern Posts (Logistics School), Xi’an University of Posts & Telecommunications, Xi’an 710061, China

**Keywords:** entropy method, TEAS, theoretical calculations, VISSIM, MADM

## Abstract

With the continuous construction of transportation infrastructure, intersection nodes have been increasing rapidly, bringing growing numbers of tunnel- and exit-adjacent sections (TEAS) in mountain expressways in China. With the complex variation in the surrounding environment, drivers always face congestion and confusion on tunnel and the exit connecting sections (TECS) without adequate length, meanwhile excessively long TECS create detours. To better provide a sustainable design strategy for TEAS, based on a certain section of expressway in Shaanxi, China, this paper establishes a theoretical calculation model through analysis. The characteristics of traffic flow and drivers’ light adaptation at tunnel exit are obtained through data collection and driving tests, and the length requirements of the tunnel and exit connecting sections (TECS) are discussed. A VISSIM microscopic simulation model is also built under various design schemes and entropy-based multi-attribute decision making (EBMADM) is used to objectively calculate the weights of the four selected evaluation indexes. Then, the design schemes of the TECS with different lengths have been comprehensively evaluated. The results show the match between the evaluation results of EBMADM with theoretical calculations under existing traffic conditions, which proves the rationality of EBMADM in such problems. For more cases, the results of the EBMADM evaluation show a positive correlation between the length of TECS for the best performing design scheme with traffic volume and diverging ratio.

## 1. Introduction

As transportation infrastructure is constantly built, a range of problems always arise. The first visible change as the expressway map densifies is the increase in intersection nodes. In mountainous areas, tunnels become important structures to connect various sections as a result of topographic constraints. In this way, many tunnel and interchange adjacent sections are produced.

In countries as mountainous as China, with such areas accounting for 69% of the total land, difficulties and challenges always abound in the construction of roads. Constrained by topography, geology and many other factors, bridges and tunnels account for a vast proportion of expressways in many mountainous areas. Tunnel- and interchange-adjacent sections are common in southern and western China, with the most common two forms being entrance ramps connecting to tunnel entrances and tunnel exits connecting to exit ramps (tunnel- and exit-adjacent sections, TEAS).

Under general conditions, diverging areas of expressways have been considered as the capacity bottlenecks of sections [1]. Drivers expecting to diverge need to recognize the exit through signs or markings and consequently change to the curb lane within a limited period and distance [2,3]. Studies also show that diversion at exits can significantly increase driver workload and errors [4,5]. Thus, vehicles that fail to prepare for the exit at the optimal moment always choose forced deceleration and lane change to remedy [6]

**Figure 1:**
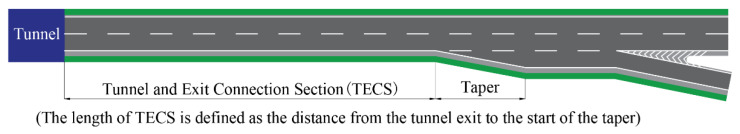
Diagram of TECS, in which complete signs typically cannot be set.

The situation frequently becomes much more complex in TEAS, with further restriction on preparation space for drivers expecting to diverge. This phenomenon is attributed to the huge difference in the light environment inside and outside the tunnel; especially so when drivers have difficulty adapting their vision in an instant to the dramatic change in illumination when exiting the tunnel, meaning it usually takes some time to recover the vision, which is called the light adaptation [7,8]. Moreover, since the narrow and dark environment of the tunnel and the disturbing effect of the cavity wall increase drivers’ discomfort, drivers changing lane in the tunnel always face a higher risk of collision [9,10]. In addition, the high severity of accidents in tunnels also prompted the prohibition of lane changes in tunnels, which means that many diverging vehicles can only change lane to exit on the short TECS [11,12]. As a result, there will be more forced lane changes and queue jumps on the TECS without adequate length, which will cause significant disruptions and congestion to traffic flow on the mainline [6,13]. Excessive congestion and queuing will further reduce the capacity of this bottleneck section, with the dissipated queue flow less than 30% of the section capacity [14,15,16]. Thus, studies also suggest that a certain clear distance should be ensured between the tunnel and the exit [17]. Figure 1 shows the coverage of TECS on TEAS. This paper defines TECS as the section from the tunnel exit to the start of the taper, since drivers are always expected to be fully prepared for diverging before the taper [3].

**Figure 2:**
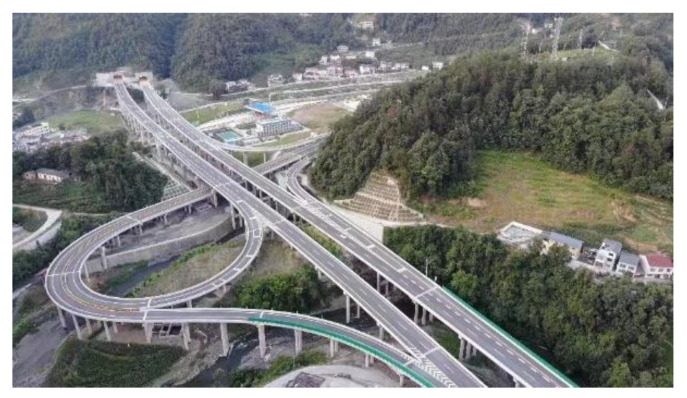
TEAS on an expressway under construction in a mountainous area, taken with an unmanned aerial vehicle (UAV) by the author, in which the town is built on the mountain.

However, on the other hand, it is also not appropriate to excessively extend the length of TECS. Ultimately, TEAS can be attributed to the special requirement of the exit location and the constraints of the terrain. Since human activity areas with exit connections are built close to the tunnel, longer TECS always result in longer detour distance. Figure 2 shows an actual example of TEAS on expressways in mountainous areas, where it can be clearly seen that the town is built near the mountain and the exit is chosen to be as close to the town as possible. Therefore, the impact of detours caused by TECS extension cannot be ignored. Thus, TECS should be designed with an appropriate length when the detours problem actually exists.

Moreover, designers should also pay attention to the increased emission of pollutants caused by congestion and detours, which has attracted significant attention with the development and deepening of the concept of sustainability [18,19,20]. The reduction in pollutants from the emission standards upgrade would be completely offset by congestion, and even heavy congestion would lead to a large increase in emissions [21,22]. Therefore, the importance of finding the optimal balance between congestion and detours has been fully highlighted.

The capacity of an expressway is frequently affected by traffic volume, geometry and traffic organization and management to some extent [23,24,25]. Some studies propose to reduce traffic demand on congested sections through charging or traffic restriction strategies to achieve traffic pressure relief [26], but others believe that a restrictive policy will not always be a good choice to face the complex transportation environment [27]. Optimization of traffic organization is also an important approach to improve the diverging areas’ capacity, including the advance arrangement of signs and dynamic management of vehicle paths [28,29]. Meanwhile, it seems that improving and upgrading road infrastructure is generally considered to be the critical solution [30,31].

Road section observation and data collection, as a traditional approach, has been frequently undertaken to support traffic analysis to draw some conclusions [32,33]. With the rapid development of numerical simulation technology, traffic simulation has become a feasible, inexpensive and effective method to evaluate a reasonable scheme, among which VISSIM is a widely recognized microscopic traffic simulation tool that has been widely used to verify the effectiveness of various traffic management strategies [34,35,36,37]. To improve the reliability of the expected evaluation results of the new scheme, the traffic simulation must realistically express driving behavior at the micro level, which can be achieved by calibration of the simulation model relying on actual traffic data [38,39].

In face of the complex transportation environment of TEAS, a more sensible approach would be to start with road infrastructure with good design strategies to improve the potential of the road itself in terms of capacity, etc. Based on the requirements of modern sustainable transportation, the evaluation of the scheme should not be limited to a single index, but rather a multi-attribute decision-making (MADM) method may be needed [40]. The entropy evaluation method has been widely used in various fields, such as physics [41], economics [42], and medicine [43], which allows a comprehensive judgment of the merits of a scheme instead of being limited to a certain index, and shows the potential of the entropy method to be applied to transportation disciplines.

With a view to fundamentally improving the constant problems on TEAS, this paper intends to explore the best design scheme of controlling length of TECS using some new methods. A theoretical length calculation model was established firstly, based on the data collection, and five different design schemes with different TECS lengths were proposed according to the result of theoretical calculation combined with the actual state of Expressway A. Subsequently, the EBMADM was applied to evaluate the result based on the VISSIM simulation calibrated with actual data, with number of vehicles, travel time, delay and CO emissions as indexes. The results of the theoretical calculations and EBMADM were validated against each other.

The rest of this article is organized as follows: Section 2 contains the engineering description and data collection, while Section 3 describes the construction of the theoretical calculation model. Section 4 includes scheme design and calibration of the simulation model, and sensitivity analysis. In Section 5, we evaluate the applicability of the scheme with the use of EBMADM and verify the validity and reliability of the method with theoretical calculation results. Conclusions are drawn in Section 6.

## 2. Engineering and Data

### 2.1. Engineering Description

This study takes a section of G69 national expressway from Ankang to Langao in Shaanxi Province, China, as an example, which is called “Expressway A” in this paper.

**Figure 3:**
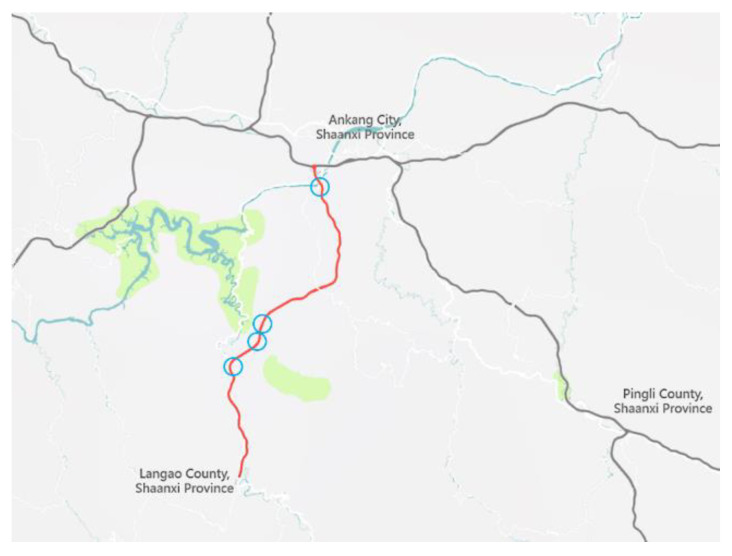
The location of Expressway A and data collection points.

Expressway A is located in the Tsinling-Daba Mountains in southern Shaanxi, with complex terrain and fluctuating peaks, at an average altitude between 1000 and 3000 m. The section is a 4-lane expressway with a speed limit of 100 km/h on the opening section of the mainline and 80 km/h in the tunnel. Due to the location, more than 42.7 km of this 47.4-km-long expressway are bridges or tunnels, which also leads to the fact that the distances between the exits of the six interchanges and the tunnels are mostly extremely short, with the shortest one only about 290 m. Figure 3 shows the location of our project, while Table 1 shows the statistics of the lengths of TECS.

The Green Book suggests that adequate decision of sight distance (DSD) should be provided to drivers facing speed, path or direction change in the interchange, with specific guidance for the value, which is about 230 m to 315 m for the design speed of 80 km/h and it will be longer for 100 km/h [44]. Since drivers cannot detect the exit inside the tunnel and it takes time for light adaptation after exiting the tunnel, TECS may require a longer length than DSD. In addition, Chinese specifications specifically provide detailed guidance on the design of TECS, with special measures suggested for TECS under 1 km in length, in which all the advance exit signs cannot be set, while interchange guidelines in China additionally propose that TECS of a two-lane expressway should preferably be no shorter than 300 m and 400 m, at a design speed of 80 km/h and 100 km/h, respectively. Therefore, the multiple TECS on Expressway A appear to be insufficient in length, especially the one at 290 m.

With increasing traffic volume, delays on the shortest TECS on Expressway A continue to increase daily during peak hours, which makes Expressway A the subject of much attention from local transportation authorities. In the author’s opinion, it is necessary and meaningful to conduct a study on such a representative section to discuss a reasonable and efficient design method of TEAC that meets the requirements of sustainable development, in which the critical focus is on the length of TECS. This paper will also investigate this issue based on the geometric design scenario and traffic case of Expressway A.

### 2.2. Data Collection

Traffic data were collected on the shorter four TECS listed in Table 1, with the locations marked by blue circles in Figure 3, as the data on the shorter TECS provide a more representative representation of the traffic characteristics of this particular section.

Data collected include traffic volume, vehicle speed and time headway on TECS, which can truly reflect the operational characteristics of traffic flow.

**Figure 4:**
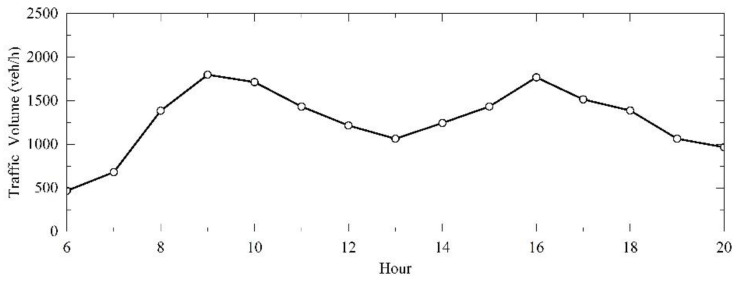
Trend of traffic volume on Expressway A, where X denotes the hour and Y denotes traffic volume on the two-lane TECS. For example, the data of “Hour 8” indicate traffic volume of both lanes on the section between 8:00 a.m. and 9:00 a.m.

Data should be collected during peak hours with good weather and no accidents or facility maintenance. Based on the preliminary research and observation, the peak hours of Expressway A frequently occur around 9:00 a.m. and 4:00 p.m. The average hourly traffic volume for a consecutive week is shown in Figure 4.

**Figure 5:**
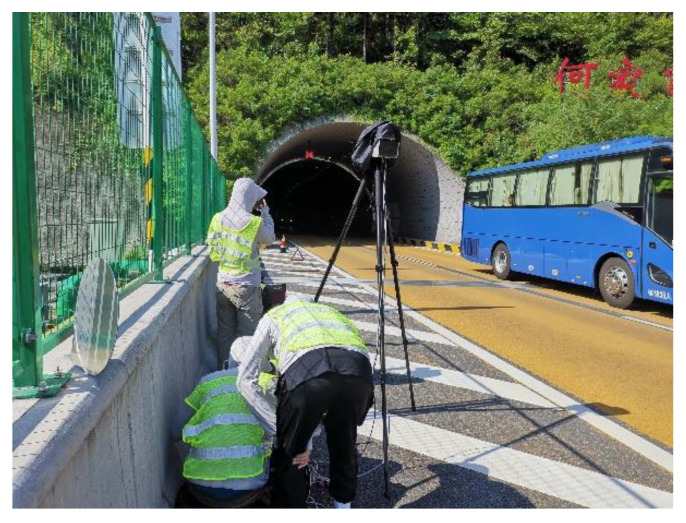
The process of data collection at the site.

**Figure 6:**
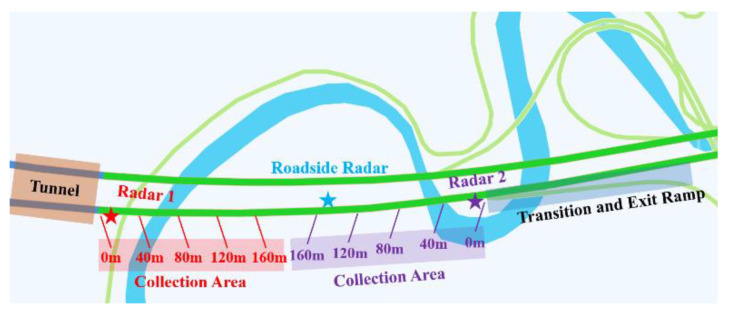
The detailed position of the data collection point on TECS. Radar 1 is set at the tunnel exit to collect vehicle speed after the tunnel, and Radar 2 is set at the start of the taper to collect vehicle speed before the taper, while the roadside radar is set 100 to 200 m before the taper to collect the time headway.

In the schedule of the data collection, two radars were placed near the exit of the tunnel and the beginning of the taper, respectively, during peak hours. At the same time, a roadside radar was placed on section 100 to 200 m from the tunnel exit, which is the position where diverging vehicles usually start to change lanes observed by the UAV. Figure 5 shows the process of data collection at the site, while Figure 6 shows the detailed location of the data collection points, and the criteria of the collected data are as follows:

- Collect all vehicle speeds during the collection period.
- Collect all vehicle types during the collection period.
- Collect all moments vehicles pass the section during the collection period.
- Collect all vehicle trajectories.

The collected data were processed in a certain procedure, mainly to reject abnormal data. Afterwards, obtained parameters such as speed, time headway, traffic volume, vehicles intensity and lane change trajectory were used to support theoretical calculation and the VISSIM model development and calibration in subsequent sections.

### 2.3. Collection Results

**Figure 7:**
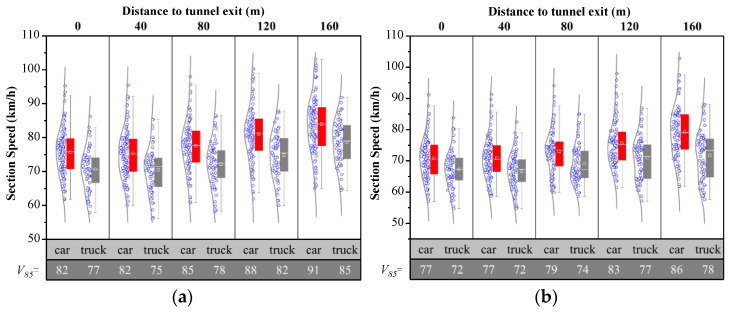
Vehicle speed after the tunnel on TECS, where X denotes the type of vehicle and distance from the tunnel exit and Y denotes vehicle speed. (**a**) Fast lane; (**b**) Curb lane.

The vehicle speed distribution was obtained by processing the data captured by the radar, while the discontinuous radar data needed to be eliminated. Figure 7 shows vehicle speed in each of the two lanes after the tunnel exit within 200 m, respectively. The average speed of cars in the fast lane around the tunnel exit is about 76 km/h, with speed ranging from 54.8 to 91.2 km/h, and after that there is an acceleration. Meanwhile, speed in the curb lane around tunnel exit ranges from 57.9 to 95.3 km/h, with the average about 71 km/h.

**Figure 8:**
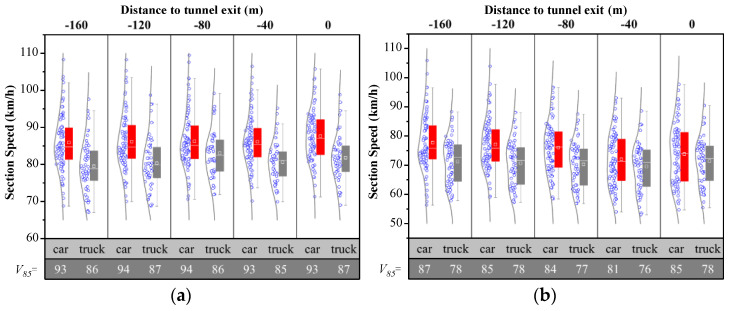
Vehicle speed before the taper on TECS, where X denotes the type of vehicle and distance from the tunnel exit and Y denotes vehicles speed. (**a**) Fast lane; (**b**) Curb lane.

Figure 8 shows vehicle speed before the start of the taper. The overall average speed inn each of the two lanes is 86.43 km/h and 75.41 km/h, respectively.

**Figure 9:**
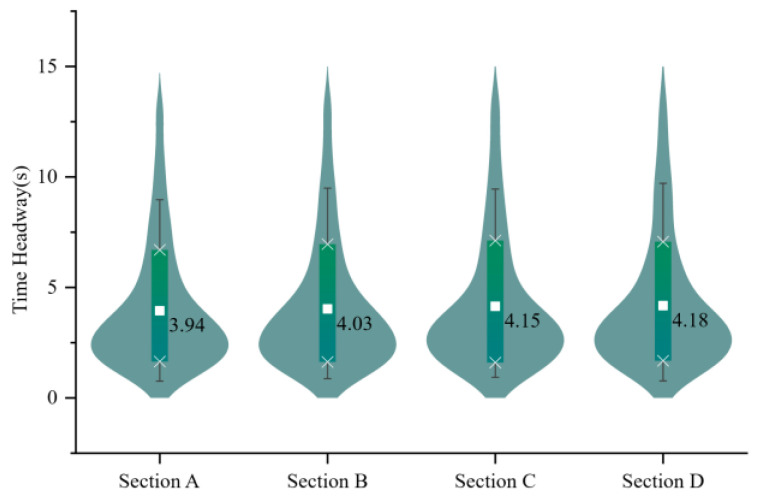
Time headway distribution in the curb lane on the TECS. Sections A to D of the X axis represent the data collection points on the four TECS in Figure 6, respectively, in the order from south to north, and Y denotes time headway on the curb lane.

The time headway data of vehicles in the curb lane which is the target lane of diverging vehicles collected by the roadside radar was selected as shown in Figure 9. Before that, abnormal data has been eliminated according to the lateral distance between vehicles and the roadside radar.

The result point to an average time headway of about 4.07 s for vehicles in the curb lane, while the fast lane is slightly less than this value, at 3.956 s. Then, vehicle intensities can be calculated from Equation (1).

(1) $$K=\frac{1000}{\bar{h_{s}}}=\frac{1000}{\bar{h_{t}}\times \bar{V}}$$

where *K* denotes the vehicle intensity in each lane, while $\bar{h_{s}}$ and $\bar{h_{t}}$ denote space headway and time headway, respectively. $\bar{V}$ denotes the average speed inn each lane.

Table 2 shows the three significant parameters inn each lane of the TECS based on collected data, which help support the conclusion that level of service (LOS) on the TECS is between LOS-B and LOS-C, as the traffic flow is stable.

This paper also collected videos of vehicles changing lanes on the TECS through UAV, which can easily determine whether the target vehicles are diverging, and used computer software to extract the vehicle trajectory data in the videos. A GPS-RTK measurement method was used to determine the coordinates of several feature points of the aerial photography section in this study, then a coordinate system was established based on this. After that, data were extracted through a YOLOv3 target detection algorithm.

Firstly, vehicles that performed the actual lane changing in the video were selected and assigned an ID, which is achieved through manual judgment. The start of the lane changing is defined as the moment when the vehicle creates a directional declination in the original lane, and the end is defined as the moment when the vehicle successfully enters the target lane and is parallel to it. Subsequently, the trained vehicle detection model was used to capture the position of the vehicle in each frame of the video, meanwhile series of data such as vehicle ID, time sequence, coordinates and speed were extracted.

**Figure 10:**
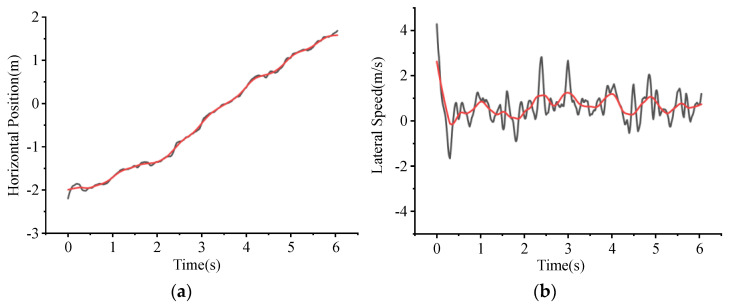
Vehicle trajectory after Kalman filter processing; the black shows the original data while the red shows the processed. (**a**) Horizontal position, where X denotes time series of lane changing and Y denotes horizontal position of the vehicle with 0 marked as the vehicle centerline coinciding with the boundary of fast and curb lane; (**b**) Lateral speed, where X denotes time series of lane changing and Y denotes the vehicle’s lateral speed, with positive values indicating the vehicle moves to the right.

The Kalman filter toolbox in MATLAB was used to smooth the extracted vehicle trajectories to eliminate errors in data extraction caused by UAV jitter in aerial photography or changes in optical elements of the video [45]. Figure 10 shows the comparison of the trajectories before and after smoothing, where the red trajectory is processed and the black is the original.

Diverging vehicles always change lanes to the right before exiting, so the trajectories of the right lane changing were processed one by one. As described above, the lateral position of each vehicle and the corresponding time can be obtained, as shown in Figure 10. The moment when the vehicle center line is at the lane-dividing line is defined as time 0 in the lane-changing process, meanwhile the abscissa of this point is also defined as 0, which makes the data presentation more intuitive and unified.

**Figure 11:**
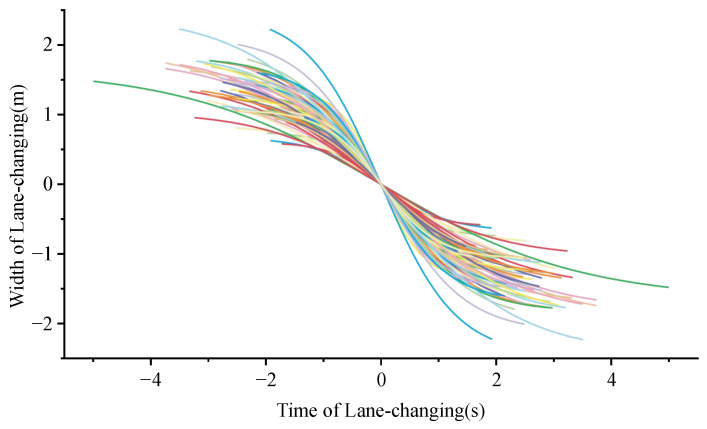
Distribution of diverging vehicle trajectory for rightward lane change. X denotes time series of lane changing with 0 marked as the moment when vehicle centerline coincides with the boundary of fast and curb lane, and Y denotes width of lane changing with 0 marked as the same means. Each line denotes a trajectory of a lane-changing vehicle.

From Figure 11, the time of lane change is distributed between 2.59 s and 9.98 s, and since the speed of each vehicle has been recorded, the distance of lane change can also be calculated.

**Figure 12:**
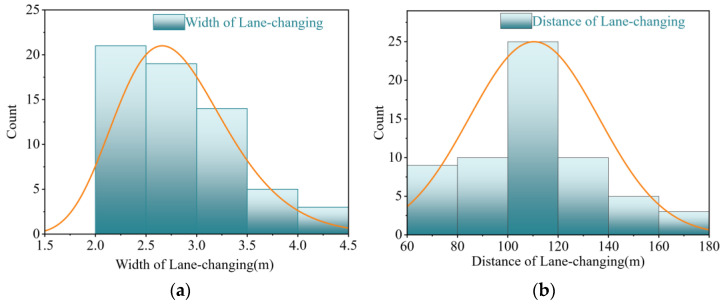
The length and width distribution of lane changing, where X denotes the width and distance of lane changing, respectively, and Y denotes the number of samples within the corresponding group. (**a**) Width of lane changing; (**b**) Distance of lane changing.

In the 71 lane-changing trajectory data, the speed distribution of vehicles is concentrated in 60~100 km/h, with the average speed of 79.96 km/h, which essentially corresponds to the data collected by our radars. The lane-changing width of most vehicles is concentrated in the range of 2 m to 4.5 m, meanwhile there are also a few data less than 2 m, which is mainly due to the long-term driving of these vehicles across lanes. Given the fact that the vast majority of vehicles are over 1.8 m wide, these 9 data would be excluded. In this way, the distribution of lane-changing length is between 65 m and 175 m, with an average of 109 m. According to statistics, the obvious difference in the lane-changing distance between private cars and trucks has not been shown. The length and width distribution of vehicle lane changing without abnormal data is shown in Figure 12.

**Figure 13:**
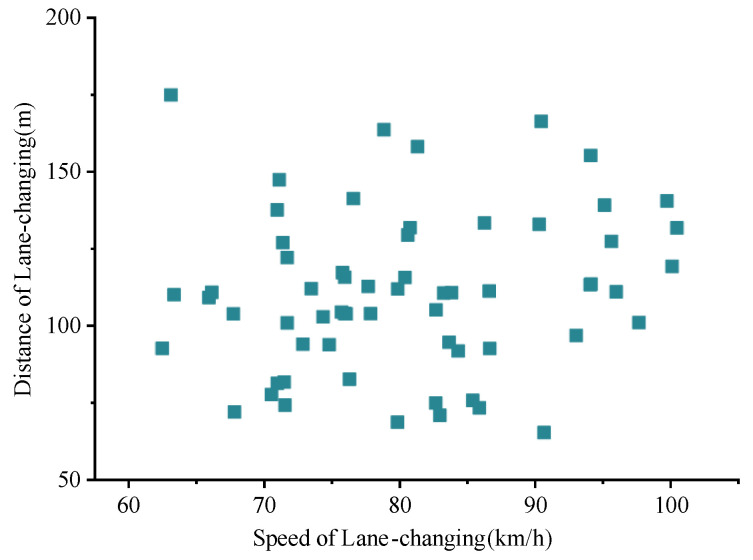
Correlation between lane-changing distance and speed. X and Y denote average speed and travel distance of the vehicle during lane changing, respectively. Each point denotes the data of a lane-changing vehicle.

Further correlation analysis was carried out on the relationship between lane-changing distance and speed, as shown in Figure 13, where a weak positive correlation can be seen. Through the correlation test, the results showed that the Pearson coefficient was 0.151, the Spearman coefficient was 0.183, and the Kendall coefficient was 0.127, which all verified our prediction results.

From the results, it can be concluded that the lane-changing distance has a certain relationship with the speed of the vehicle, but it also reflects a reality that the lane-changing distance also has a certain relationship with the driver’s skill. Drivers who choose high speeds usually have a higher skill level, which weakens the correlation with speed. Therefore, taking 80 km/h as the boundary, the lane-changing distance was counted separately, and the results are shown in Table 3.

### 2.4. Experiment

The experiment was carried out by recruiting volunteers to drive on the exit section of the tunnel, aiming to obtain dynamic data on the pupil area of the driver when exiting the tunnel. The experiment was also carried out on the Expressway A, and the experimental period when the traffic flow was relatively stable was selected as much as possible, which aimed to minimize the interference with the data results. Considering that studies have shown that drivers’ visual adaptation process is related to strong changes in light intensity, the experiment was selected to be carried out at noon in clear weather [46,47].

**Figure 14:**
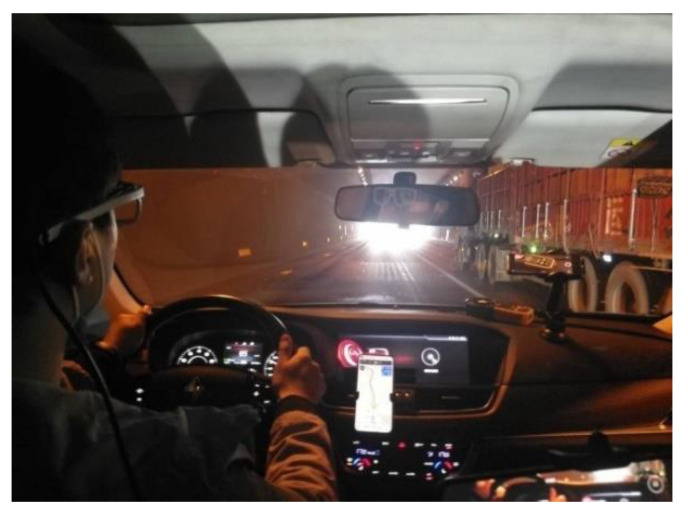
Driving experiments on the section with drivers wearing SMI ETGTM.

24 volunteers aged from 22 to 55 with 3 to 28 years of driving experience were required to wear SMI eye-tracking glasses (SMI ETG) to drive in sequence, which can track pupil trajectory and characteristics of the driver and record data including pupil size and gaze position and time, and are widely used in studies of light adaptation [48]. Another person recorded the vehicle speed and illumination data at the corresponding moment, which is shown in Figure 14.

**Figure 15:**
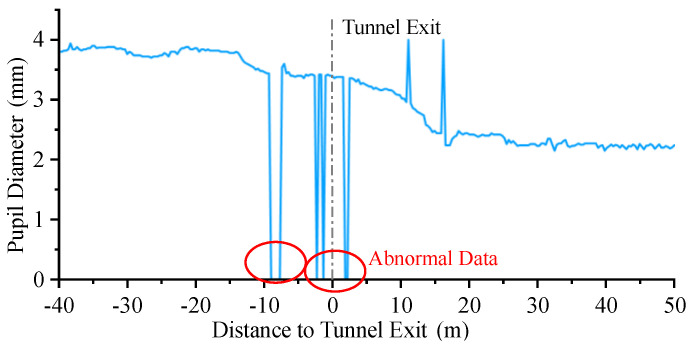
Normal and abnormal data of driver pupil diameter., where X denotes the distance from a point to tunnel exit, and Y denotes driver’s pupil diameter at this position.

It was found that there were some data with pupil diameter of 0 in the compilation of the data, which is shown in Figure 15. These cases were mainly caused by the drivers’ involuntary blinking, while the failure of the SMI ETG to capture drivers’ pupil data due to bumps is also a reason for consideration.

Typically, there is minimal difference between the pupil diameters of human each eye. Based on this, we can use the non-zero pupil diameter to replace the other pupil diameter that is displayed as 0, but data with both pupil diameters of 0 can be eliminated.

The change in the pupil area of human beings when the light environment changes is a normal physiological phenomenon, so research refers to the concept of heart rate turbulence in medicine to introduce pupil oscillation to evaluate the degree of visual discomfort of drivers when exiting the tunnel [49]. The beginning of pupil oscillation is defined as the moment when the pupil area is greater than 50% of the previous moment, while the end is interpreted as the moment when the pupil area shrinks to 50% of the previous moment.

**Figure 16:**
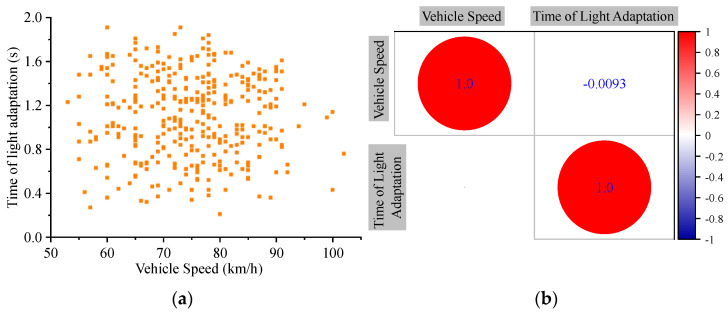
The distribution of the time of light adaptation. (**a**) Light adaptation results distribution, where X denotes speed of the vehicle at tunnel exit, and Y denotes light adaptation time for the driver, each point denotes the data of a driver; (**b**) Correlation of vehicle speed with light adaptation time, where the value denotes the correlation between factors corresponding to X and Y.

The vehicle speed distribution in the experiment is between 53 to 102 km/h, which is the speed of most vehicles on TECS in data collection, and most of the samples have the phenomenon of visual secondary oscillation with an interval of about 1 s. Therefore, the time of light adaptation can be interpreted as the time from the beginning of the first oscillation to the end of the last oscillation. By arranging groups of data with a total sample size of 352, the distribution of the time of light adaptation in Figure 16a was obtained.

To better provide precise evidence for the subsequent research in this paper, the effect of vehicle speed on time of light adaptation also needs to be discussed. Since a precise conclusion cannot be drawn directly from Figure 16a, correlation analysis in statistical method was used to discuss the relationship between speed and time of light adaptation. The Spearman correlation coefficient is a nonparametric index that measures the dependence of two variables with a monotonic equation. With Results ranged in [−1, 1], the larger absolute value represents the higher correlation [50].

Figure 16b also shows the results of the Spearman correlation analysis of the adaptation time and vehicle speed, only the correlation coefficient of −0.0093 between the two shows their weak correlation. Such a result may be attributed to the interference from other influences, as studies have shown that drivers’ visual characteristics near tunnel exits are related to a variety of factors, not only from environmental aspects such as differences in illumination between the inside and outside of the tunnel, but also from drivers’ own physiological differences such as age, gender and adaptation ability. Thus, the same time of light adaptation is taken for different speeds, but without taking the average value of 1.11 s, since the authors believe that the 85th percentile will satisfy more drivers; refer to the explanation of operating speed in the Green Book [44]. The maximum is not adopted in order to avoid the effect of experimental errors. Based on this, 1.53 s is suggested as the time of light adaptation for drivers exiting the tunnel in the research.

## 3. Model Development of Theoretical Calculation

### 3.1. Analysis

According to the analysis of drone aerial video, it can be intuitively detected that almost all vehicles do not change lanes immediately after getting out of the tunnel. Instead, more drivers will stay in the original lane for a while before driving to the curb lane, which is mainly related to the actual time demand on drivers to go through the process of light adaptation and determine the exit.

**Figure 17:**
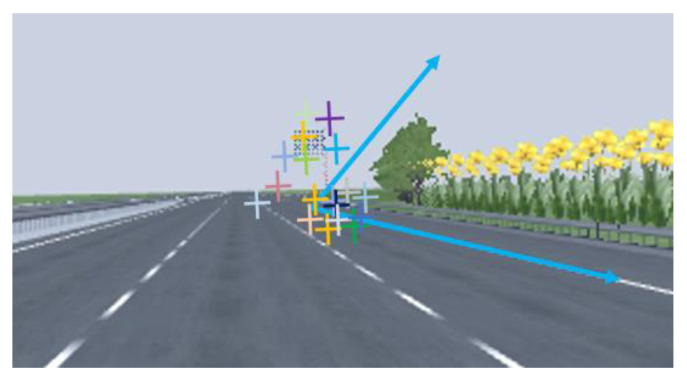
Drivers’ gaze position to identify the exit ramps in the simulation driving test.

On TECS, drivers usually need to undergo the light adaptation at first, after which, drivers will take different actions in the following two situations. In one case, drivers can directly see the exit ramp when their vision returns to normal. According to a questionnaire we conducted with some drivers, more than half of them identified exit ramps through spotting a change in the marking line at the beginning of the taper, while some research indicates that this distance should be around 400 m [51]. Our research proved it through a simulation driving test, as shown in Figure 17.

According to this, we define the first case as when the distance from the tunnel exit to the beginning of the taper is less than 400 m, and name that Case A. In Case A, drivers will find the exit by recognizing the change in marking line when their vision returns to normal, and make decisions to change lanes to enter the ramp.

In the other case, when the distance is more than 400 m, which we call Case B, drivers cannot directly detect the existence of the exit. As usual, they choose to accelerate to adapt to the mainline after leaving the tunnel, but perhaps an advance guidance sign can help avoid this step. In this way, the drivers’ behavior after visual restoration will include reading the sign, making decision and changing lanes to leave the mainline.

Preferably the vehicles will already be in the outmost lane, so they can exit without changing lane, which means that drivers only need to discover the existence of the exit ramp after visual restoration. Certainly, another case is that drivers already know the location of the exit when they are in the tunnel, so they can change lanes at the right place after leaving the tunnel. Of course, the process of visual restoration is still necessary. This situation can be achieved by setting guidance sign in advance, although currently it is rare to set it in the tunnel.

### 3.2. Distance Control

**Figure 18:**
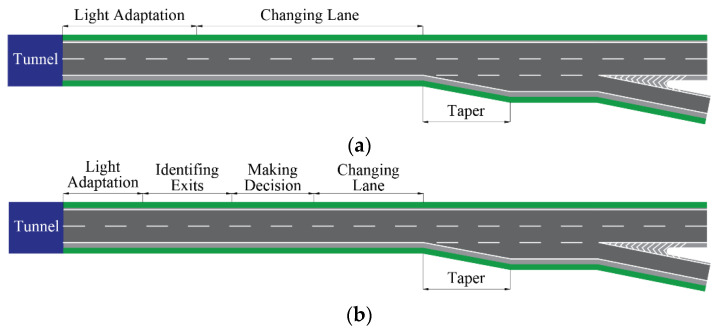
Components of the connection section. (**a**). Case A and Case B; (**b**). Case C.

Based on the above description, we can divide the connection section between the tunnel and the exit into several modules, as shown in Figure 18. The connection section is defined as the section from the exit of the tunnel to the beginning of the taper, which is consistent with the result of drivers’ gaze position in our experiment. How to identify exits becomes the main difference between Cases A and B, and we also consider the case when the sign is set in advance as a reference for the minimum length limit of the TECS when conditions are extremely difficult, which is named Case C.

### 3.3. Distance Advice

In view of the fact that the average speed of private cars is significantly higher than that of trucks, while significant differences have not been shown in other aspects, considering the unfavorable situation, the distance control of private cars is analyzed. In the adaptation stage, according to the results of the data investigation and experiment, the average speed of private cars in the fast lane near the tunnel exit is approximately 76 km/h, and the driving distance is about 32 m with a light adaptation time of 1.53 s.

It takes about 1.5 to 2 s for drivers to see the signs or markings on the expressway, referring to the value of the discovery time in the sight distance of AASHTO [44]. In this way, the distance traveled is approximately 42 m at a speed of 76 km/h.

Research has shown that the decision time is related to the amount of information required for the decision, as shown in Equation (2) [52].

(2) $$t_{2}=1.237554e^{0.258913x}$$

where *t*_2_ denotes the decision time and *x* denotes the amount of information. Drivers need to decide whether or not to exit the mainline, which includes 1 bit of information, therefore, it takes 1.6 s to make the decision, corresponding to a driving distance of 34 m.

From a microscopic point of view, the lane changing of the vehicle is affected by the insertable clearance in the target lane [53]. Research shows that the time headway distribution of the vehicle in the mainline can obey the K-order Erlang distribution [54], negative exponential distribution [55], Cowan M3 distribution [56], or GEV distribution [57]. Through SPSS data analysis, the measured data are used to fit these models, and it can be concluded that the time headway of the research target road section approximately obeys the third-order Erlang distribution.

However, when the third-order Erlang distribution is applied to the single-lane time headway representation, there is a large time headway between 0 and 1.2 s, which is inconsistent with the actual situation. In order to improve this kind of error, the distribution curve is moved to the right along the *t*-axis by a minimum value of time headway, and the modified third-order Erlang distribution curve can be obtained. Probability of time headway greater than *t* can be calculated as shown in Equation (3).

(3) $$P(h\geq t)=(4.5\lambda^{2}{\left(t-\tau \right)}^{2}+3\lambda (t-\tau )+1)e^{-3\lambda (t-\tau )}$$

where λ denotes the average arrival rate of vehicles per unit time, which can be calculated by λ=Q/3600 and τ denotes the minimum value of time headway, which is considered to be 0.76 from the survey results.

Thus, the corresponding probability density in Equation (4) can be obtained.

(4) $$f\left(t\right)=13.5\lambda^{3}{\left(t-\tau \right)}^{2}e^{-3\lambda \left(t-\tau \right)}$$

The condition that the headway distance on the target lane is greater than or equal to the minimum space headway *t_c_* must be met to successfully change lanes, otherwise the space headway will be rejected. In this way, the queuing time for the insertable clearance can be averaged through the theory of probability and statistics, as shown in Equation (5).

(5) $$t_{w}=\frac{\int_{\tau }^{t_{c}}tf\left(t\right)dt}{1-P\left(h\geq t_{c}\right)}\times \frac{1-P\left(h\geq t_{c}\right)}{P\left(h\geq t_{c}\right)}=\frac{\int_{\tau }^{t_{c}}tf\left(t\right)dt}{P\left(h\geq t_{c}\right)}$$

where *t_w_* denotes the queuing time for the insertable clearance and *t_c_* denotes the critical minimum clearance for vehicles, which can be referred to the value of the 1 m/s for lane-changing traverse rate in the Japanese Road Construction Order [58].

The survey has shown that the average speed of the fast lane (target lane) approach to the exit is about 77 km/h, and the peak traffic volume on the connecting section is about 900 pcu/h. Approximately 1.6 s becomes the average time for an insertable gap to appear on the target lane according to calculations, which can also be considered as the average queuing time of drivers in the process of lane- changing, corresponding to a driving distance of 34 m. The survey results show that the lane-changing distance is distributed between 65 to 175 m, which is related to the drivers’ skill and the urgency of changing lanes [59]. Thus, the average speed of 113 m above 80 km/h can be taken as the approximate demand, which corresponds to a lane-changing time of about 45 s.

In this way, an ample space for drivers to leave the mainline uses a value ranging between 3.0 and 9.1 s in Cases A and B, that is to say, a connection section more than 260 m can fit the demand. In Case C, this value can be reduced to 180 m, which means an 8 s travel.

## 4. VISSIM Simulation

### 4.1. Scheme Design

In the simulation schedule, the four TECS schemes in Expressway A corresponding to Case A (length of TECS less than 400 m) were selected, which are also the main targets of data collection. Meanwhile, considering the length gap between the different schemes, a shorter TECS with a length of 250 m, slightly less than the theoretical calculation results, was taken into simulation, which can be used for the validation of the theoretical calculation results. These schemes are named as Scheme 1–Scheme 5 according to the length of TECS from smallest to largest.

As shown in the above results, it is clearly not appropriate to change lane on the exit section of tunnel where drivers need to adapt to light. Therefore, lane changing should be prohibited here in the VISSIM model. Subsequently, in Case A, diverters would not change lane immediately because of their need to read signs and make a decision, while others may still be free to do this. This feature can be reflected in the vehicle path and behavior settings of VISSIM. Thus, the schemes for simulation can be designed as shown in Table 4.

According to the results from the data collection, the traffic volume and diverging ratio on TECS are listed. Trucks account for nearly 30% of the traffic volume of about 1800 veh/h on the two-lane TECS, and the diverging ratio of the four interchanges is between 20% and 25%. The two are also added as variables in the study. Considering traffic volume and diverging ratio of Expressway A for the design target (3000 veh/h and less than 50% respectively for a two-lane section), more detailed traffic cases for simulation are formulated, as shown in Table 5. The total traffic volume starts from 1800 veh/h, which approximately reflects traffic volume at the current stage of Expressway A, and extends to its design traffic volume of 3000 veh/h, with a gradient of 300 veh/h. Meanwhile, two additional cases of 3300 veh/h and 3600 veh/h are also taken into the simulation to explore more cases. The diverging ratio, on the other hand, takes 10% and 40% as the boundary, with a gradient of 10% divided into four groups.

According to the data collection, vehicles in the fast lane account for about 50.73% of the total vehicles on the section around the tunnel exit, which is also reflected in our simulation to restore a more realistic traffic flow. The diverging ratio for each lane was not set precisely to ensure its randomness, based on the observation of measured data. As a result, 28 cases representing the majority of traffic conditions have been developed.

### 4.2. Calibration of the VISSIM Simulation Model

The accuracy of the VISSIM simulation model should be calibrated to make it closer to the actual situation. The following parameters need to be entered into the VISSIM model according to the standard calibration procedure proposed in previous studies:

- Geometric parameters of the TECS and the exit of the interchange were used.
- Through and diverging traffic volumes were 1363 and 431 vehicles at peak on the TECS, respectively, according to the data collection, and the diverging ratio was 24%.
- Vehicle speed range on the TECS was 53.1 to 109.5 km/h.
- The percentages of trucks and cars on the TECS were 27% and 73%, respectively.

The high sensitivity of the capacity reflects the accuracy of the VISSIM model, which was selected as the calibration index of this paper among several commonly used indexes. The capacity is calculated by Equation (6).

(6) $$C=\frac{3600}{\bar{h_{t}}}$$

where *C* denotes the ideal capacity (veh/h) and $\bar{h_{t}}$ denotes the average minimum time headway (s).

The mean absolute percent error (MAPE), as an index which reflects the discrepancy between the simulated and collected capacities, can be calculated by Equation (7).

(7) $$MAPE=\frac{\sum_{i=1}^{n}C_{v}^{i}-\sum_{i=1}^{n}C_{f}^{i}}{\sum_{i=1}^{n}C_{f}^{i}}$$

where $C_{v}^{i}$ denotes the simulated capacity in the VISSIM model (veh/h), $C_{f}^{i}$ denotes the investigated capacity, *i* denotes traffic flow and *n* denotes a total of through and diverging traffic flows.

Table 6 shows the calculated MAPE with the results of −2.51%, which represents the capacity error of the VISSIM model to reality, and it can be accepted in practical engineering applications [60].

### 4.3. Evaluation Indexes Selection

As usual, the evaluation of design method covers several aspects including capacity, traffic safety and environment. Among them, travel time, delay and number of stops are the most commonly used evaluation indexes in VISSIM microsimulation evaluation [61]. Meanwhile, number of vehicles, CO, NOx, VOC emissions and fuel consumption can also be added to the node results in VISSIM.

Among them, delay is an important index to measure the smoothness of traffic operation, while number of vehicles directly reflects the capacity of a section. Furthermore, TEAS occur mainly because residences, factories or other human activity areas are located in close proximity to tunnels, as well as road construction constraints. Thus, longer TECS always brings more detours, and since the detour time after exiting the highway cannot be measured with different specific conditions, the travel time of vehicles on TECS is selected as another evaluation index, which can indirectly reflect the results on the time spent from congestion and detours. Lastly, CO emissions, as an important index to measure the operation performance of the section in environmental protection, are also selected, which not only show the pollutant emissions level of the vehicles on the section, but also indirectly reflect fuel or other energy consumption.

Based on comprehensive consideration, number of vehicles, travel time, delay and CO emissions are selected as evaluation indexes in this paper to evaluate the impact of design method with different lengths of the TECS on various aspects.

### 4.4. Sensitivity Analysis

Sensitivity analysis was carried out on simulation test results of five schemes, which reflects the improvement of each scheme compared with Scheme 1 (250 m as the length of the connection section, marked “L = 250 m”) The whole sensitivity analysis includes improvement ratio in each of the four selected indexes for Schemes 2–5 compared to Scheme 1, with each comparison covering results of the 28 traffic cases set in VISSIM simulation. 

#### 4.4.1. Scheme 2 Compared with Scheme 1

**Figure 19:**
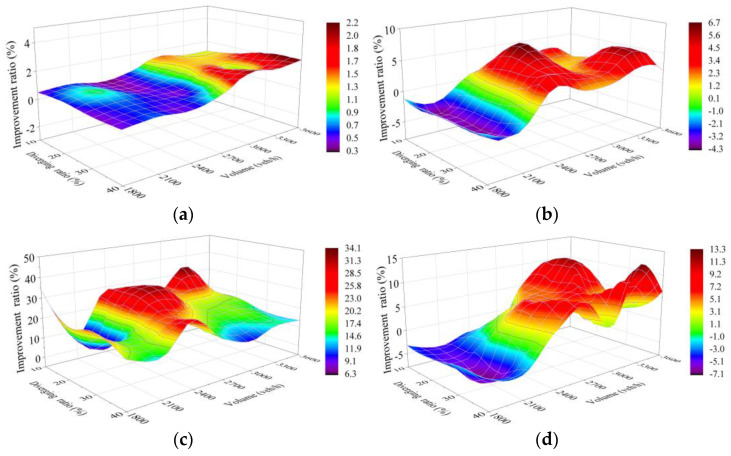
Improvement ratio of Scheme 2 (L = 290 m) compared with Scheme 1 (L = 250 m). (**a**) Capacity; (**b**) Travel time; (**c**) Delay; (**d**) CO emissions. X and Y denote the total traffic volume and diverging ratio on the two-lane TECS, respectively, while Z shows the improvement ratio of Scheme 2 compared with Scheme 1 based on simulation results for the two schemes in each case. The label on the right side shows the values represented by each color to help with reading.

Figure 19 shows the improvement for Scheme 2 (L = 290 m) compared with Scheme 1. Since, of the four selected indicators, a high number of vehicles is the one that is more expected to be achieved, while the other three (travel time, delay time and CO emissions) are expected to be lower, the improvement ratio of number of vehicles should be calculated according to “Ratio = 100% × (Scheme 2 − Scheme 1)/Scheme 1” and the ratio of the other three should be calculated according to “Ratio = 100% × (Scheme 1 − Scheme 2)/Scheme 1”.

As shown in Figure 19a, the improvement ratio of number of vehicles of Scheme 2 approaches 0 with the traffic volume lower than 2700 veh/h on TECS, whereas with the further increase in traffic volume and diverging ratio, the improvement holds a trend of increasing, but with the maximum only at 2.3%.

Figure 19b shows a negative improvement ratio of travel time for low traffic volume and diverging ratio, which reflects the fact that vehicles experience longer travel time on the TECS in Scheme 2. This phenomenon should likely be analyzed in conjunction with the variation trend of delays in Figure 19c. The delay improvement peaks at the traffic volume from 2400 to 2700 veh/h in Scheme 2, with a maximum ratio of 32%, which can be clarified as the delay in low level with the low traffic volume in these two schemes, while it will likely be difficult for the TECS under 290 m to meet the smooth demand with the high traffic volume. Clearly, the travel time is directly related to the length of the TECS and the delay. Therefore, the travel time would be longer in Scheme 2 with the traffic volume lower than 2400 veh/h. At 2700 veh/h, the improvement ratio reaches a peak of nearly 10%, and shows a more constant trend at larger traffic volumes.

Figure 19d reflects the improvement ratio of CO emissions. Similar to fuel consumption, CO emissions are correlated with travel time and driving speed, while stopping and restarting also further increase fuel consumption and CO emissions. Although traffic flow on the TECS of Scheme 2 operates more smoothly, the non-negligible increase in detour distance makes CO emissions worse at the low traffic volume and merging ratio, which is shown in the figure as a negative improvement ratio. Positive improvement in emissions is also shown with the better numerical improvement under the heavier traffic, which completely offsets the effect of detours. Therefore, the trend of CO emission improvement under different conditions reflected in the figure is similar to the travel time in Figure 19b, but with the difference in CO emissions affected by vehicle speed and stopping.

#### 4.4.2. Scheme 3 Compared with Scheme 1

Similar to the above comparison, the improvement ratio of Scheme 3 (L = 320 m) to Scheme 1 for number of vehicles should be calculated by “Ratio = 100% × (Scheme 3 − Scheme 1)/Scheme 1”, while that for the other three should be calculated by “Ratio = 100% × (Scheme 1 − Scheme 3)/Scheme 1”.

**Figure 20:**
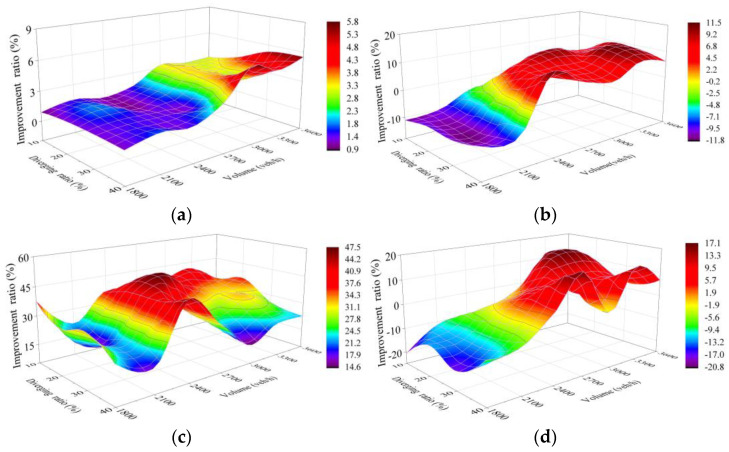
Improvement ratio of Scheme 3 compared with Scheme 1. (**a**) Capacity; (**b**) Travel time; (**c**) Delay; (**d**) CO emissions. X and Y denote the total traffic volume and diverging ratio on the two-lane TECS, respectively, while Z shows the improvement ratio of Scheme 3 compared with Scheme 1 based on simulation results for the two schemes in each case. The label on the right side shows the values represented by each color to help with reading.

The trend of number of vehicles improvement ratio in Scheme 3 reflected in Figure 20a shows similarity to that in Scheme 2, but shows a better effect, with a maximum of 5.8%.

Figure 20c shows the average improvement ratio, which reaches more than 30%, and the peak, which reaches 47.4%, in terms of delay in Scheme 3, both of which are better than the performance of 19.6% and 34% in Scheme 2. The improvement also gets a great optimization when the traffic volume is higher than 2700 veh/h and the diverging ratio is lower than 30% on the TECS synchronously.

This change is also reflected in travel time and CO emissions as shown in Figure 20b, the improvement in travel time is optimized because of the further optimization of the improvement in delay under heavy traffic. However, the improvement ratio is negative with the light traffic conditions, which means a longer travel time. This phenomenon can also be explained as the fact that the improvement of low delays cannot compensate for the increased travel time from longer detour distance in Scheme 3 in this traffic environment. Similarly, some changes in CO emission improvement are also associated with this, as shown in Figure 20d.

#### 4.4.3. Scheme 4 and Scheme 5 Compared with Scheme 1

**Figure 21:**
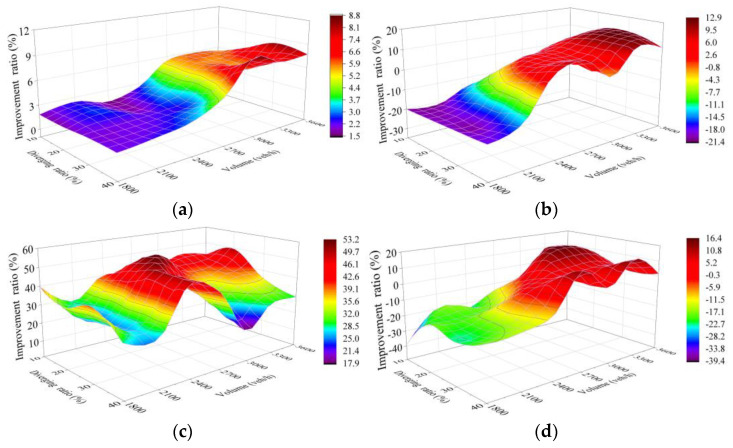
Improvement ratio of Scheme 4 compared with Scheme 1. (**a**) Capacity; (**b**) Travel time; (**c**) Delay; (**d**) CO emissions. X and Y denote the total traffic volume and diverging ratio on the two-lane TECS, respectively, while Z shows the improvement ratio of Scheme 4 compared with Scheme 1 based on simulation results for the two schemes in each case. The label on the right side shows the values represented by each color to help with reading.

**Figure 22:**
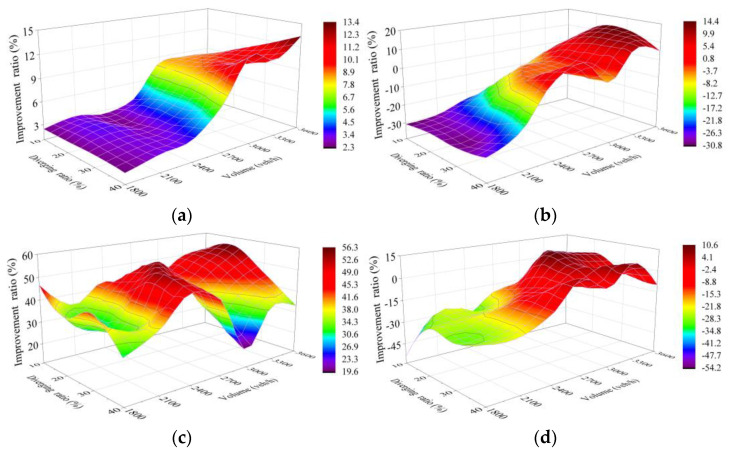
Improvement ratio of Scheme 5 compared with Scheme 1. (**a**) Capacity; (**b**) Travel time; (**c**) Delay; (**d**) CO emissions. X and Y denote the total traffic volume and diverging ratio on the two-lane TECS, respectively, while Z shows the improvement ratio of Scheme 5 compared with Scheme 1 based on simulation results for the two schemes in each case. The label on the right side shows the values represented by each color to help with reading.

With the improvement ratio calculated in the same way as the above two comparisons, Figure 21a and Figure 22a show the number of vehicles improvement of Scheme 4 and Scheme 5, respectively, in which the trend is similar to the previous two schemes. The better performance is reflected in the results, with peaks of 8.8% and 13.4% and averages of 4.3% and 6.2%, respectively.

Figure 21b and Figure 22b reflect the worse performance of improvement in travel time under the low traffic volume to diverging ratio in the scheme with a longer length of TECS, with the lowest being −19.9% and −30.7%, respectively, which is related to the fact that a large proportion of improvements in low delays under this condition are still slight. However, the schemes with longer connection sections show better improvement in travel time, which contradicts the earlier appearance of the peak of improvement ratio in Scheme 2, with the traffic volume above 3000 veh/h and the diverging ratio of 20–30% on the TECS. Subsequently, the travel time improvement ratio also shows a downward trend under the higher diverging ratio, which can be analyzed in combination with the rule of delay improvement.

Figure 21c and Figure 22c show the improvement ratio in delay of Scheme 4 and Scheme 5, respectively, which achieve the optimum (49.5% in Scheme 4 and 56% in Scheme 5) with a larger traffic volume and a lower diverging ratio. However, the improvement still remains to be optimized under the higher diverging ratio, which also indicates demand for further increase in the length of the TECS under this condition. Meantime, this phenomenon corresponds to the decrease in travel time improvement ratio at a diverging ratio of more than 40% under heavy traffic volume.

## 5. Evaluation on Entropy Method

According to the improvement of the five schemes in 28 cases (Figure 19, Figure 20, Figure 21 and Figure 22), it is difficult to find the optimal scheme directly, due to the fact that each of the five schemes possessed its own advantages. As a consequence, MADM is necessary to evaluate the specific benefits of these five schemes. Based on this context, entropy method, which scientifically and effectively calculates the index weight according to the amount of information provided by the index observation value, is an appropriate choice.

Entropy is a measure of uncertainty in information theory. When the amount of information is larger, the uncertainty is smaller, resulting in less entropy, and vice versa. Based on its characteristics, the entropy value can be used to evaluate the dispersion degree of a certain index, with a greater degree of dispersion of an index indicating greater impact of the index on the comprehensive evaluation.

To sum up, the tool of information entropy was utilized to calculate the weight of each index based on the degree of variation of the four indicators selected, which provides a basis for MADM and grades the simulation results of each case to rank the five schemes.

### 5.1. Weight of Each Indexes

As shown in Equation (8), a matrix with five rows and four columns is built based on the simulation results of the five schemes in each case, in order to compare the index differences among these schemes in 28 cases.

(8) $$A_{k}={\left[\begin{array}{cccc} V_{1,k} & T_{1,k} & D_{1,k} & C_{1,k}\\ V_{2,k} & T_{2,k} & D_{2,k} & C_{2,k}\\ V_{3,k} & T_{3,k} & D_{3,k} & C_{3,k}\\ \begin{array}{l} V_{4,k}\\ V_{5,k} \end{array} & \begin{array}{l} T_{4,k}\\ T_{5,k} \end{array} & \begin{array}{l} D_{4,k}\\ D_{5,k} \end{array} & \begin{array}{l} C_{4,k}\\ C_{5,k} \end{array} \end{array}\right]}_{5\times 4}$$

where *V*_1*,k*_ denotes the number of vehicles of Scheme 1 in the case *k*, while *T*_1,*k*_, *D*_1,*k*_ and *C*_1,*k*_ denote travel time, delay and CO emissions in the corresponding case, respectively.

The weight in each *A_k_* needs to be calculated based on entropy method, as each weight of the four indicators of the 28 groups of simulation results is different. Firstly, *y_ij_* is utilized as each *A_i,k_* in the matrix *A_k_* to denote *A_k_* as *Y*.

(9) $$Y={\left[\begin{array}{cccc} y_{11} & y_{12} & y_{13} & y_{14}\\ y_{21} & y_{22} & y_{23} & y_{24}\\ y_{31} & y_{32} & y_{33} & y_{34}\\ \begin{array}{l} y_{41}\\ y_{51} \end{array} & \begin{array}{l} y_{42}\\ y_{52} \end{array} & \begin{array}{l} y_{43}\\ y_{52} \end{array} & \begin{array}{l} y_{44}\\ y_{54} \end{array} \end{array}\right]}_{5\times 4}$$

*y_j_* denotes each column of matrix *Y*.

(10) $$y_{j}={\left[y_{1j},y_{2j},y_{3j},y_{4j},y_{5j}\right]}^{T}$$

Then, matrix Y can be converted into the following form:

(11) $$Y=\left[y_{1},y_{2},y_{3},y_{4}\right]$$

where *y*_1_, *y*_2_, *y*_3_ and *y*_4_ denote number of vehicles, travel time, delay and CO emissions, respectively, of the five schemes. The larger number of vehicles indicates the better scheme, while the other three are the opposite. Thus, the four indexes must be forward processed, so that higher values of the three indexes indicate better schemes, which can unify the evaluation method, as shown in Equation (12)

(12) $$y_{1j}{}^{\prime }=\left\{ \begin{array}{l} \max\left\{y_{1j},y_{2j},y_{3j},y_{4j},y_{5j}\right\}-y_{1j},j\neq 1\\ y_{11},j=1 \end{array}\right.$$

In this way, the matrix Y’ is obtained by the forward processing.

(13) $$Y^{\prime }=\left[y_{1}{}^{\prime },y_{2}{}^{\prime },y_{3}{}^{\prime },y_{4}{}^{\prime }\right]$$

where *y_j_*′ denotes the *y_j_* after forward processing.

(14) $$y_{j}{}^{\prime }={\left[y_{1j}{}^{\prime },y_{2j}{}^{\prime },y_{3j}{}^{\prime },y_{4j}{}^{\prime },y_{5j}{}^{\prime }\right]}^{T}$$

Subsequently, all values are required to be standardized to convert absolute indexes into relative indexes to solve the problem of homogeneity of different qualitative indexes, which is calculated as in Equation (15).

(15) $$y_{ij}^{\prime \prime }=\frac{y_{ij}^{\prime }-\min\left\{y_{1j}^{\prime },y_{2j}^{\prime },y_{3j}^{\prime },y_{4j}^{\prime },y_{5j}^{\prime }\right\}}{\max\left\{y_{1j}^{\prime },y_{2j}^{\prime },y_{3j}^{\prime },y_{4j}^{\prime },y_{5j}^{\prime }\right\}-\min\left\{y_{1j}^{\prime },y_{2j}^{\prime },y_{3j}^{\prime },y_{4j}^{\prime },y_{5j}^{\prime }\right\}}$$

The weight of index *j* in the case *k* can be calculated as in Equation (16).

(16) $$p_{ij}=\frac{y_{ij}{}^{\prime \prime }}{\sum_{i=1}^{n}y_{ij}{}^{\prime \prime }},i=1\;to\;5,j=1\;to\;4$$

Equation (17) shows the entropy calculation of exponential *j*.

(17) $$e_{j}=-k\sum_{i=1}^{n}p_{ij}\ln\left(p_{ij}\right)$$

where k=1/ln(n) and satisfies $e_{j}\geq 0$.

The redundancy of information entropy can be calculated.

(18) $$d_{j}=1-e_{j}$$

Then the weight of each index can be calculated using Equation (19).

(19) $$\omega_{j}=\frac{d_{j}}{\sum_{j=1}^{m}d_{j}}$$

The weight matrix *W_k_* denoting four indexes under the traffic volume group *k* can be calculated through the above process, and the results are shown in Table 7.

(20) $$W_{k}=\left[w_{k,1},w_{k,2},w_{k,3},w_{k,4}\right]$$

In each case, the specific score of each index of each scheme and the total score of each scheme can be calculated by Equations (21) and (22), respectively.

(21) $$z_{ij}=w_{ij}\times p_{ij},\;j=1\;to\;4$$

(22) $$z_{i}=\sum_{j=1}^{4}z_{ij}$$

### 5.2. Evaluation Result and Discussion

**Figure 23:**
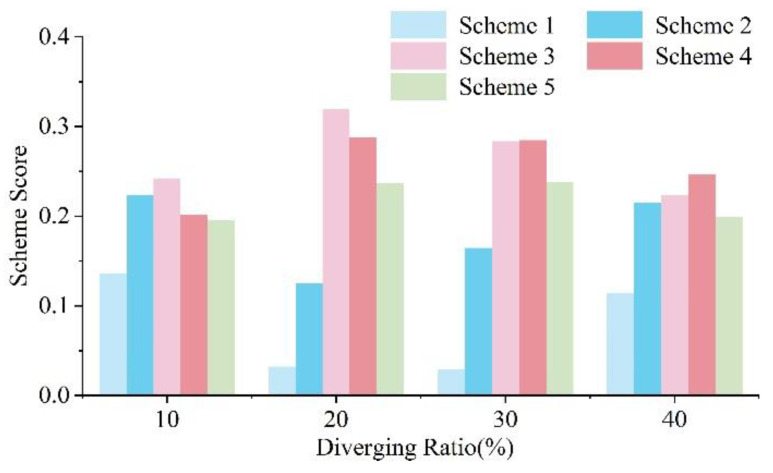
Design schemes score for a two-lane TECS at 3000 veh/h traffic volume. X denotes traffic cases with different diverging ratios and Y denotes the comprehensive score of the scheme in the corresponding case. Scheme 3 performs the best with a diverging ratio less than 20%, while Scheme 4 becomes the optimal scheme with that above 30%.

Following the above steps, a specific score for each scheme in each traffic case can be calculated based on the simulation result. Figure 23 shows the scores for each scheme with the traffic volume of 3000 veh/h on the two-lane TECS, which is expected to be reached on Expressway A in the future.

**Figure 24:**
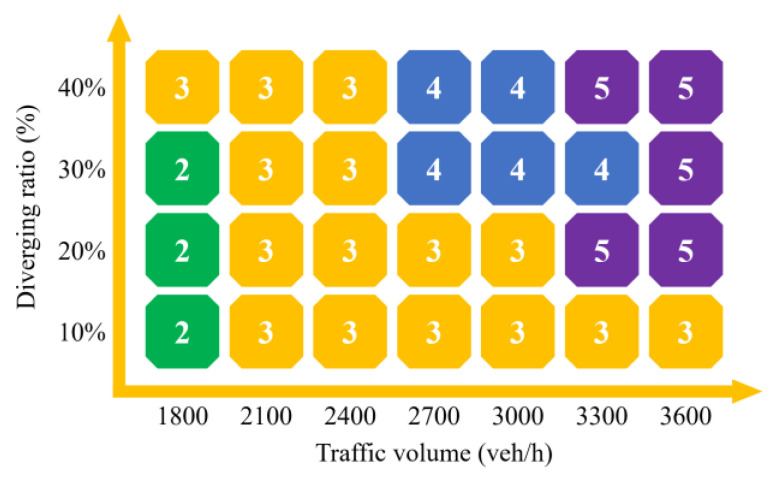
The optimal design scheme based on the results of EBMADM. X denotes traffic volume on the two-lane TECS and Y denotes diverging ratio. The optimal scheme for each of the 28 traffic cases has been shown.

According to the results, the length of the TECS is expected to be 320–360 m based on the current diverging ratio of 20–25% at each exit of Expressway A. In addition, the final scores for each of the five schemes in 28 cases were calculated, and the design scheme with the best performance in each case is selected, as shown in Figure 24.

Scheme 1 (L = 250 m) does not appear on the recommendation list of any case considered in this paper, which indicates that a 250 m long TECS failed to meet the smooth requirements in these common traffic environments.

Scheme 2 (L = 290 m) takes up the bottom three blocks on the left, which represent the current traffic situation on Expressway A, meaning that Scheme 2 is the optimal choice under the traffic volume of about 1800 veh/h while the diverging ratio is less than 30%. This result also reflects another fact: the conclusion that 260 m as the shortest length of the TECS in the existing traffic environment of Expressway A obtained by theoretical calculation is reasonable. Conversely, the matching results also verify the accuracy of the entropy method.

Scheme 3 (L = 320 m) takes up the majority of blocks in the lower left triangle with traffic volume below 2400 veh/h or diverting less than 20% of traffic, and leads the recommendation list. The TECS length of 290 to 320 m in Schemes 2 and 3 is only close to the DSD in the Green Book, and even slightly inadequate after subtracting the distance of light adaptation about 30 m, and it is also less than the 400 m recommended for the section with the design speed of 100 km/h by Chinese specifications. However, most of their applicable cases do not exceed 2400 veh/h, which is less than the 3000 veh/h traffic volume applicable to the Chinese specifications for a two-lane expressway. Considering the fact that vehicles do not have to queue for too long to change lanes at low traffic volumes, such a result is actually acceptable, referring to the theoretical calculation result at a traffic volume of 1800 veh/h in this paper.

However, the performance of Scheme 4 (L = 360 m) is better than the others under the traffic volume higher than 2400 veh/h and the diverging ratio higher than 20%. Another boundary occurs at 3000 veh/h; traffic volume higher than this requires a longer TECS to ensure a smooth traffic environment (Scheme 5, L = 400 m, or a much longer connection section, based on the sensitivity analysis in the previous chapter which shows more room to optimize the delay improvement under high traffic volume and diverging ratio, which can be researched further), with the diverging ratio more than 20%.

The results of EBMADM show the optimal length of TECS for different traffic cases, which considers the detour impact caused by TECS extension. When detours can be disregarded or do not exist, the optimal length of TECS in Figure 23 can be transformed into the minimum length, since the difference in TECS length between schemes is not large, only 3040 m, and the detour distance is actually acceptable, so when the negative impact of the detour is greater than the improvement in traffic efficiency and emissions due to the extension of TECS, this improvement has actually saturated.

To sum up, the volume of traffic plays a key role in the selection of the length of the TECS. Especially in the case of low traffic volume, the decision between Scheme 2 and Scheme 3 almost takes the volume of traffic as its dividing line. Of course, the diverging ratio also plays an important role, especially under traffic volume exceeding 2700 veh/h, when the diverging ratio directly determines whether a longer TECS option is desirable. This phenomenon can be further analyzed in association with theoretical models. The key variation is still the different result of different traffic conditions as vehicles change lanes and intertwine before the exit. Vehicle lane changes often require longer queuing times and distances under higher traffic volume, while similar results can be caused as a consequence of more turbulent traffic flow in the diverging area under the higher diverging ratio.

To summarize, the results of EBMADM provide a certain basis for the planning and design of the engineering, which enables effective guiding of the engineering to the target of sustainable development under specific, but common, cases. In other words, in the specific engineering design of TECS, the predicted design traffic volume and diverging ratio, which can be obtained through the traffic survey in the engineering feasibility study, can be an appropriate basis for selecting the length of TECS. It contributes to a green design scheme that maximizes traffic efficiency and reduces carbon emissions.

## 6. Conclusions

Rapid construction of expressways in mountainous areas of China creates more and more TEAS. The short TECS constrained by construction conditions always aggravates the capacity bottleneck in diverging areas of exits, and leads to excessive delays and emissions. Meanwhile, the TECS with excessive length also create longer detour distances. Thus, this paper provides an EBMADM method to evaluate different design schemes for TECS with different length in different traffic cases, which is supported by the simulation result of the VISSIM model built and calibrated based on the actual measured data. The theoretical calculation result is taken to verify the accuracy of EBMADM, while Expressway A supports the study as an example of engineering. Conclusions drawn from this study are as follows:

- The traffic volume and diverging ratio on the TECS determines its optimal length.
- The TECS with a length of 300 m is the appropriate design with traffic volume less than 2400 veh/h, which is between LOS-A and LOS-C, while with the traffic volume and diverging ratio increasing to above 2700 veh/h and 20%, respectively, the TECS needs to be extended to 350–400 m.
- The results of EBMADM show more negative impact in traffic efficiency and emissions than positive impact from detour reduction with the actual length of TECS less than the optimal length. Meanwhile, however, since the detour distance is not actually significant, typically less than 100 m, the optimal length of TECS can be considered as the minimum length when detours can be accepted.
- The theoretical calculation results supported by the measured data validate the accuracy of EBMADM. For the TECS with traffic volume of 1800 veh/h and diverging ratio of 20%, theoretical calculation shows that the TECS with length of 260 m can support vehicles diverging, which is close to the 290 m suggested by EBMADM.
- In the specific engineering design, the TECS length suggested by EBMADM can be taken as a reference for different traffic volume and diverging ratio, which can be acquired from the traffic survey in the engineering feasibility study.
- Although all TECS of Expressway A can support relatively stable traffic in the current traffic conditions (volume of 1800 veh/h), for the design target volume of 3000 veh/h, some TECS may face heavy congestion in the future with the growth of traffic. Therefore, some better solutions need to be investigated further.

Restricted to page space, only one representative road section is studied in this paper. Since more and more TEAS appear, prior to using this method in the future, some issues should be studied, including and not limited to: 1. Whether the accuracy can continue to be guaranteed in the case of more lanes since this paper only studied the case of two lanes on the mainline. 2. Whether the adjacent section of the mainline entrance and the tunnel entrance, which also serve as sections with significant potential hazards, can continue to be studied with this method. The authors will focus on these issues in future studies.

## Figures and Tables

**Table 1:** Statistics on the length of TECS on Expressway A.

Group	Interchange (Exit)	Tunnel	Length of TECS
1	Z	L	670 m
2	J	L	450 m
3	S	Q	320 m
4	S	H	290 m
5	T	T	400 m
6	L	V	360 m

**Table 2:** The three significant traffic parameters on the TECS.

Lane	Speed (km/h)	Volume (veh/h)	Intensity (veh/km)
Fast lane	82.31	910	11.06
Curb lane	75.19	884	11.75

**Table 3:** Statistics on the lane-changing distance.

Lane-Changing Speed (km/h)	60 to 80	80 to 100
Lane-changing Distance (m)	65 to 166	68 to 175
Average (m)	108	113

**Table 4:** The schemes designed for simulation.

Scheme	Light Adaptation	Reading Signs	Making Decision	Queuing and Lane-Changing	Length of TECS
1	32 m	42 m	34 m	142 m	250 m
2				182 m	290 m
3				212 m	320 m
4				252 m	360 m
5				292 m	400 m

**Table 5:** The further detailed traffic cases for simulation.

Case	1	2	3	4	5	6	7
Total Volume (veh/h)	1800	2100	2400	2700	3000	3300	3600
Volume on the fast lane (veh/h)	913	1065	1217	1370	1522	1674	1826
Volume on the curb lane (veh/h)	887	1035	1183	1330	1478	1626	1774
Case	1	2	3	4			
Diverging Ratio (%)	10%	20%	30%	40%			

**Table 6:** MAPE index calculation results.

Flow	Through Traffic	Diverging Traffic
Investigated Capacity (veh/h)	1417	377
Simulated capacity (veh/h)	1385	364
Individual MAPE (%)	−2.26	−3.45
MAPE (%)	−2.51	

**Table 7:** Description of weight calculation results for each traffic case.

Index	Average Weight for 28 Cases	Weight Range
Traffic Volume	0.3086	[0.2479, 0.3691]
Travel Time	0.2409	[0.1901, 0.3193]
Delay	0.2129	[0.1675, 0.2623]
CO emissions	0.2376	[0.1902, 0.3036]

## Data Availability

Not applicable.
